# Bite Marks

**DOI:** 10.1007/s10912-024-09889-3

**Published:** 2024-08-22

**Authors:** Lorna Sankey

**Affiliations:** University of East Anglia, Norwich, UK

Sandra had left me a piece of cheese on a little saucer in the bathroom. What an odd place to leave it, I thought. She was doing increasingly strange things recently. She’d always been a bit different though, ever since she was small.

I took a bite but it tasted a bit off. She probably hadn’t checked the use by date. I spat it into the toilet and flushed it away.

“Sandra” I shouted. “The cheese has gone off.”

She appeared at the door, looking flustered. I wish she’d try to relax more.

“Mum, my name’s not Sandra. How many times do I have to….”

She looked down at the little saucer.

“Mum! Tell me you haven’t eaten the soap!”

I told you she was going barmy.

“Of course I haven’t eaten the soap. Anyway, when’s lunch?”

“We finished lunch 20 minutes ago mum.”

She was frantically searching the bathroom for something. She looked in the cupboard over the sink, in the bin, behind the loo rolls.

“Have you lost something love?”

“Mum, tell me you haven’t eaten the soap. Open your mouth. Let me see.”

She tried to part my lips but I backed away. The humiliation. Maybe I’d ring the doctor and ask him to see her.

“Oh, mum, what are we going to do with you….”

I looked down and saw that she’d put a little saucer next to the sink. On it was a piece of cheese that she’d taken a bite out of.

“Sandra, why on earth are you eating cheese in here? You need to take a break love. Why don’t you go and lie down.”

